# Glycolytic activity is required for the onset of neural plate folding during neural tube closure in mouse embryos

**DOI:** 10.3389/fcell.2023.1212375

**Published:** 2023-07-03

**Authors:** Daisuke Sakai, Yuki Murakami, Daichi Shigeta, Mitsuhiro Tomosugi, Hiromi Sakata-Haga, Toshihisa Hatta, Hiroki Shoji

**Affiliations:** ^1^ Department of Biology, Kanazawa Medical University, Uchinada, Ishikawa, Japan; ^2^ Department of Hygiene and Public Health, Kansai Medical University, Osaka, Japan; ^3^ Department of Anatomy, Kanazawa Medical University, Uchinada, Ishikawa, Japan

**Keywords:** hypoxia, neural tube closure, metabolism, glycolysis, mouse

## Abstract

Physiological hypoxia is critical for placental mammalian development. However, the underlying mechanisms by which hypoxia regulates embryonic development remain unclear. We discovered that the expression of glycolytic genes partially depends on hypoxia in neuroepithelial cells of E8.25 mouse embryos. Consistent with this finding, inhibiting glycolysis during the early phase of neural tube closure (E8.0–8.5) resulted in a neural tube closure defect. In contrast, inhibiting the electron transport chain did not affect neural tube formation. Furthermore, inhibiting glycolysis affected cell proliferation, but not differentiation and survival. Inhibiting glycolysis repressed the phosphorylation of myosin light chain 2, and consequent neural plate folding. Our findings revealed that anaerobic glycolysis regulates neuroepithelial cell proliferation and apical constriction during the early phase of neural tube closure.

## Introduction

Placental mammalian embryos are exposed to rapid changes in environmental oxygen and nutrient availability during development (Fischer and Bavister, 1993; Leese, 1995; Dunwoodie, 2009; Ufer and Wang, 2011). The oxygen concentration is relatively high in the oviduct (∼8%) where fertilization and pre-implantation development occur, but ∼3%–5% in the uterus where blastocyst implantation occurs. Furthermore, post-implantation embryos encounter hypoxic conditions as they become embedded in the endometrium (Ufer and Wang, 2011). Therefore, mammalian embryos are considered to acquire and develop an adaptive system for extreme changes in oxygen concentration. The conventional ablation of hypoxia-inducible factor 1 α (*Hif1α*), an oxygen-dependent transcriptional activator, causes defective cardiovascular formation, somitogenesis, and neural tube closure, resulting in embryonic lethality (Iyer et al., 1998; Ryan et al., 1998; Kotch et al., 1999; Lee et al., 2001; Compernolle et al., 2003; Ream et al., 2008). Furthermore, hypoxia is necessary for normal early post-implantation development in rodent embryos cultured *ex utero* (Morriss and New, 1979). These findings suggest that post-implantation embryos express hypoxia-inducible genes that regulate the metabolism, angiogenesis, and other cellular functions necessary for gastrulation and organogenesis.

Several types of mammalian cells under normoxia rely on aerobic respiration *via* the tricarboxylic acid (TCA) cycle and electron transport chain (ETC) to generate ATP. In contrast, cells alter their metabolic dependence to anaerobic glycolysis *via* Hif1α-dependent hypoxia signaling under hypoxic conditions (Semenza, 2003). To increase anaerobic glycolysis, Hif1α activates its target glycolytic genes, glucose transporter 1 (*Glut1*), hexokinase 2 (*HKII*), lactate dehydrogenase A (*Ldha*), aldolase A (*Aldoa*), and 6-phosphofructo-2-kinase/fructose-2,6-biphosphatase 3 (*Pfkfb3*). In early mammalian development, energy metabolism is rewired for developmental progression in embryos at the stage of neural tube closure (NTC), depending on the increased oxygen supplied by maternal blood (Miyazawa et al., 2017; Miyazawa et al., 2018; Fame et al., 2019; Fame and Lehtinen, 2021). Mouse embryos exhibit metabolic plasticity in response to the changes in oxygen concentration during the early phase of the NTC stage at embryonic day (E) 8.5 but not E7.5 (Miyazawa et al., 2018). Thus, anaerobic glycolysis appears to be exclusively activated in mouse embryos before E8.5, and glucose metabolism is gradually shifted to the TCA cycle, ETC, from E8.5, depending on increases in environmental oxygen concentrations (Miyazawa et al., 2017; Miyazawa et al., 2018). In addition, the activation of glycolysis regulates neural crest cell delamination under physiological conditions (Bhattacharya et al., 2020). Collectively, these findings suggest the essential roles of glucose metabolic shift in embryogenesis.

Mouse embryos at E8.5 cultured with Carbonyl cyanide p-trifluoro-methoxyphenyl hydrazine (FCCP), an uncoupler of the ETC, that disrupts ATP synthesis, causes NTC defect (Miyazawa et al., 2018). This finding suggests that glucose metabolism functions in NTC. Furthermore, inhibiting glycolytic enzyme activity leads to severe defective neural tube formation (Hunter and Tugman, 1995). However, the cellular mechanisms by which glycolytic enzymes regulate NTC remain poorly understood.

This study discovered that glycolytic genes are expressed in the neural plate (NP) and induced by hypoxia in mouse embryos. Furthermore, glycolytic activity is required for the apical constriction of neuroepithelial cells. This is essential for NP folding, which is an early step in NTC. Inhibiting glycolysis moderately reduced neuroepithelial cell proliferation. Our findings showed that glycolytic enzymes localized at the apical surface. Thus, anaerobic glycolysis might be required for local ATP production to promote NP folding and cell proliferation during NTC in mouse embryos.

## Results

### Glycolytic genes are expressed in neural plates of mouse embryos

**FIGURE 1:**
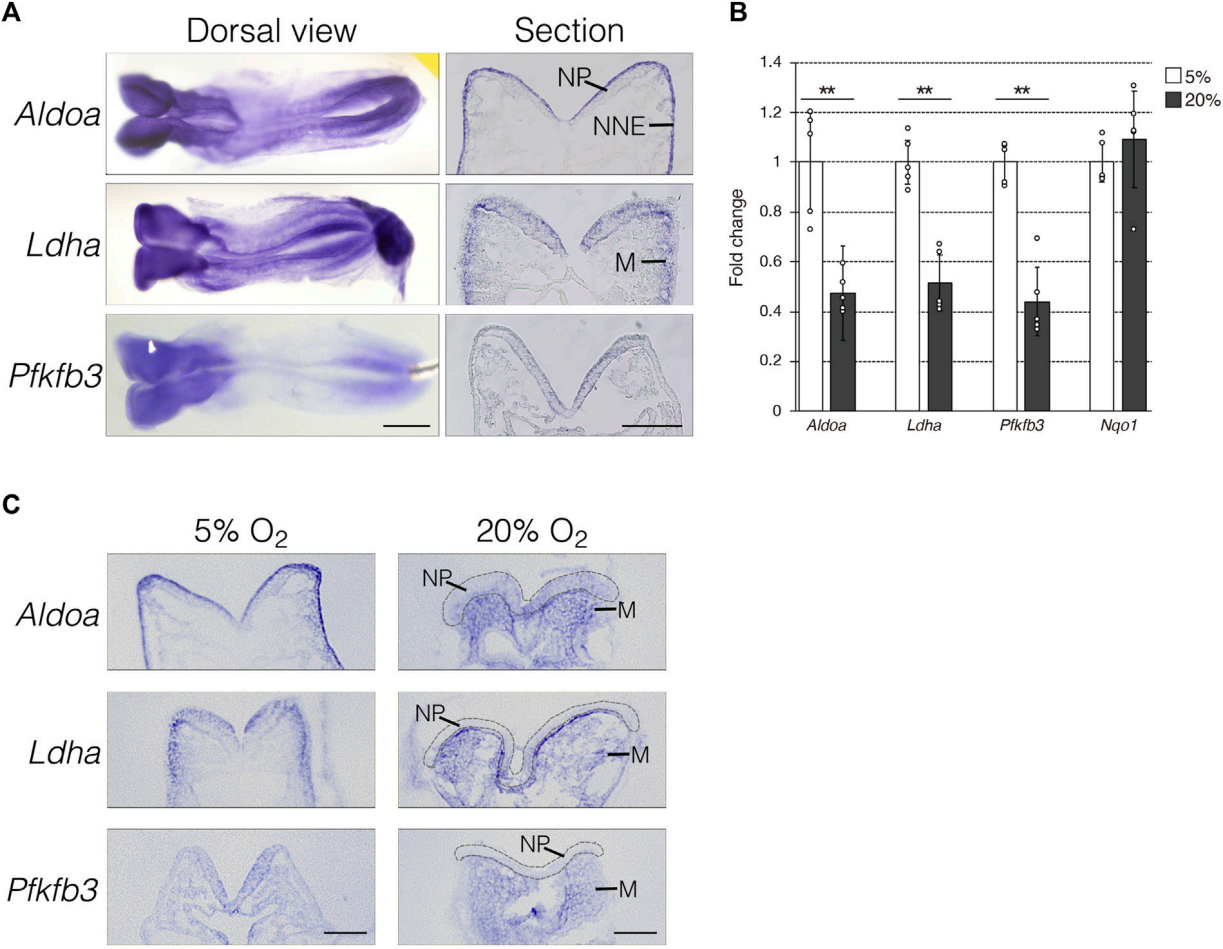
Hypoxia-dependent expression of glycolytic genes in the mouse embryo. **(A)** Expression of *Aldoa, Ldha,* and *Pfkfb3* mRNA was detected in whole-mount (Dorsal view) and cryosection (Section) *in situ* hybridization of E8.25 (six to eight somite stage) embryos. Scale bars, 500 μm (Dorsal view) and 100 μm (Section). Data are representative of six independent experiments. NP, neural plate; NNE, non-neural ectoderm; M, cranial mesenchyme. **(B)** Expression of *Aldoa, Ldha, Pfkfb3,* and *Nqo1* after the whole-embryo culture with 5% or 20% O_2_ determined by RT-qPCR. Messenger RNA levels of genes were normalized to that of *Gapdh*, and the relative values are presented as white (5% O_2_) and gray (20% O_2_) bars. Data are shown as means ± S.E.M of five embryos. Statistical differences were assessed using Student’s *t*-tests, **p < 0.001. **(C)** Embryos were cultured under hypoxia (5% O_2_) or normoxia (20% O_2_) from E8.0 (three to five somite stage) to E8.25 (six to eight somite stage). Expression of *Aldoa, Ldha,* and *Pfkfb3* mRNA was detected by *in situ* hybridization. Neural plates are indicated by gray dotted lines. Scale bars, 100 μm. Data are representative of four independent experiments. NP, neural plate; M, cranial mesenchyme.

To determine the role of anaerobic glycolysis in NTC, we initially confirmed the mRNA expression of the hypoxia-inducible glycolytic genes *Aldoa*, *Ldha*, and *Pfkfb3* using whole-mount *in situ* hybridization. The expression of *Aldoa*, *Ldha*, and *Pfkfb3* was abundant in the anterior and posterior NP of E8.0 (three to five somite stage) mouse embryos (Figure 1A, dorsal view). Next, we analyzed the mRNA expression profiles using transverse sectioning. We found that *Aldoa* mRNA localized to the apical surface of neuroepithelial and non-neural ectodermal cells, *Ldha* mRNA was expressed in neuroepithelial and mesenchymal cells adjacent to the non-neural ectoderm, and *Pfkfb3* mRNA was detected in neuroepithelial cells (Figure 1A, section). Thus, our finding revealed that NP expression was common among these three genes. We examined whether the expression of glycolytic genes is dependent on hypoxia. We developed E8.0 (3-5 somite stage) mouse embryos under normoxia (20% O_2_) or hypoxia (5% O_2_) and then isolated RNA at E8.5 (9–11 somite stage). The results of RT-qPCR revealed that glycolytic gene expression decreased by ∼ 50% under normoxia compared with hypoxia (Figure 1B). The expression of NAD(P)H dehydrogenase (quinone) 1 (*Nqo1*) that encodes the electron respiratory chain enzyme is independent of, and was not altered by hypoxia. These results indicated that glycolytic genes are expressed in NP and that this part depends on hypoxia. Furthermore, we determined whether hypoxia-dependent glycolytic gene expression is tissue-specific. To address this, we performed *in situ* hybridization (Figure 1C). *Aldoa* mRNA expression was diminished in the apical surface of neuroepithelial and non-neural ectodermal cells by normoxia. Unexpectedly, *Aldoa* mRNA turned out to be expressed in cranial mesenchyme of embryos cultured under normoxia. *Ldha* mRNA expression was significantly reduced in neuroepithelial cells, but slightly increased in cranial mesenchyme by normoxia. Furthermore, *Ldha* mRNA was highly expressed in cranial mesenchymal cells localized near the basal side of NP. *Pfkfb3* mRNA expression was observed in embryonic cranial mesenchyme, but not in neuroepithelial cells under normoxia (Figure 1C). Collectively, these findings demonstrated that glycolytic gene expression is dependent on hypoxia in neuroepithelial cells of early stage embryos.

### Glycolytic enzyme activity is essential for the early phase of NTC

**FIGURE 2:**
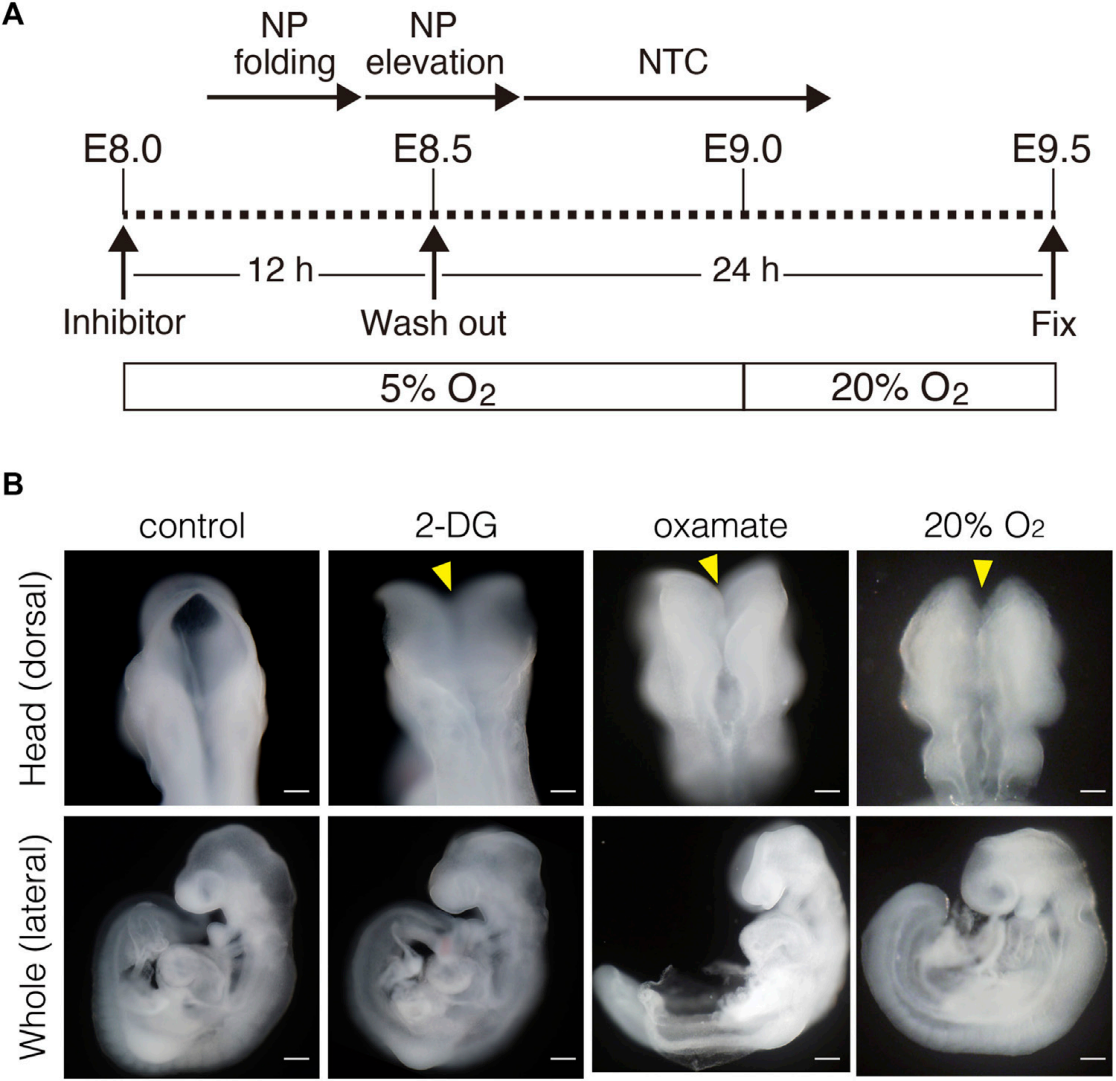
Inhibition of glycolytic activity results in neural tube closure defects. **(A)** Schema of *exo utero* whole-embryo culture with glycolysis inhibitors. Cellular events occurring during the neural tube closure process are shown above. NP, neural plate; NTC, neural tube closure. **(B)** Morphology of head and whole body of embryos incubated with PBS (control), 0.1 mM 2-DG (2-DG), 28 mM oxamate (oxamate), and normoxia (20% O_2_). Yellow arrowheads, defective neural tube closure. Scale bars, 100 μm (head), 500 μm (whole). Images are representative of 33 (control), 21 (2-DG), 15 (oxamate), and 8 (20% O_2_) embryos after *exo utero* whole-embryo cultur.

**FIGURE 3:**
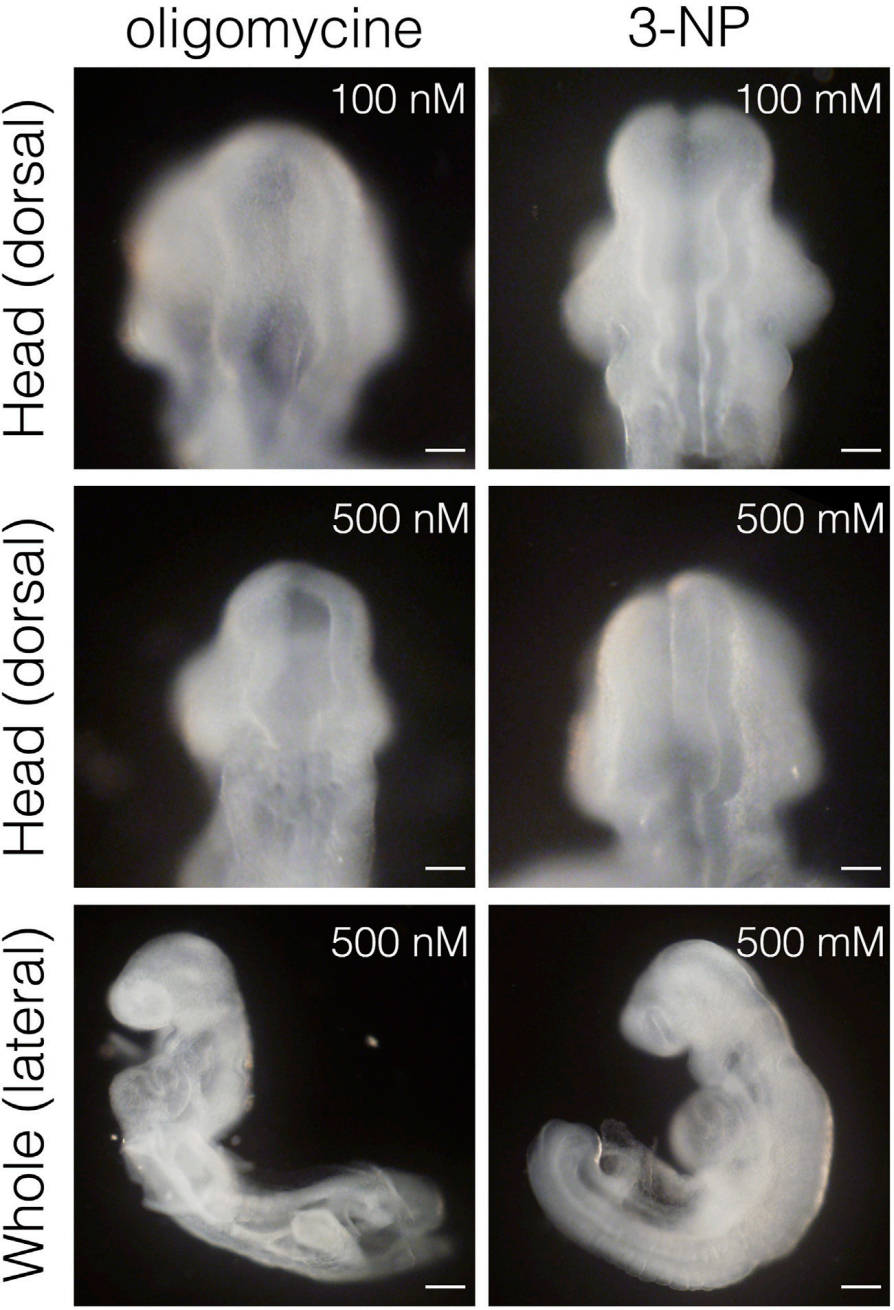
Inhibition of ETC, results in severe growth retardation, but not neural tube closure defect. Head and whole body morphology of embryos incubated with oligomycin and 3-NP. Scale bars, 100 μm (head), 500 μm (whole). Images are representative of 23 (oligomycin) and 20 (3-NP) embryos after *exo utero* whole-embryo culture.

Inhibiting glycolysis using the glucose analog 2-deoxy-D-glucose (2-DG) prevents NTC (Hunter and Tugman, 1995). Therefore, we reevaluated the role of glycolytic enzyme activity in NTC. First, we confirmed that inhibiting glycolysis prevented NTC in our *ex utero* whole-embryo culture system. E8.0 (3-5 somite stage) embryos were cultured with 2-DG for 24 h, then the gross morphology of the embryos was analyzed. We found that NTC was completely impaired by 2-DG, whereas untreated embryos formed normal NTs (data not shown). Next, we incubated E8.0 (3-5 somite stage) embryos with 2-DG or the lactate dehydrogenase inhibitor sodium oxamate (oxamate) for 12 h, then removed the inhibitors by changing the culture medium. The embryos were cultured for a further 24 h (Figure 2A). Figure 2A also shows the developmental events that occurred during *exo utero* whole-embryo culture. Neural tubes formed normally in control cultures (81.2%, 27/33 embryos). In contrast, NTC was completely prevented by the glycolytic inhibitors (2-DG; 100%, 21/21 embryos. Oxamate; 100%, 15/15 embryos), whereas developmental defects were not found in other tissues (Figure 2B, yellow arrowheads). Incubation with glycolytic inhibitors for 12 h (E8.0-8.5, 3–11 somite stage) was sufficient to prevent NTC, suggesting that glycolytic activity is required for the early phase of NTC process. Moreover, NTC is prevented by *exo utero* whole-embryo culture under normoxia (Figure 2B, 20% O_2_) (75.0%, 6/8 embryos). This result shows that a hypoxic condition is required for glycolytic gene expression during embryogenesis. The TCA cycle and ETC, are the major metabolic pathway for ATP production after E8.5. Hence, incubating E8.5 embryos with FCCP causes NTC defects (Miyazawa et al., 2018), indicating that the ETC, is involved in the late phase of the NTC. We examined if incubation with an ETC, inhibitor for 12 h (E8.0–8.5) affects NTC progression. To examine this, E8.0 embryos (3-5 somite stage) were incubated with the ATP synthase inhibitor oligomycin, or the electron transport chain complex II inhibitor 3-nitropropionic acid (3-NP). Oligomycin and 3-NP obviously retarded embryonic growth and caused abnormal gross morphology, whereas NTC was not impaired in oligomycin-treated (74.0%, 17/23 embryos) and 3-NP-treated embryos (80.0%, 16/20 embryos) (Figure 3). These results indicated that the ETC, is not required during the early phase of NTC progression, at least during E8.0-8.5.

### Glycolytic activity is required for the onset of NP folding

**FIGURE 4:**
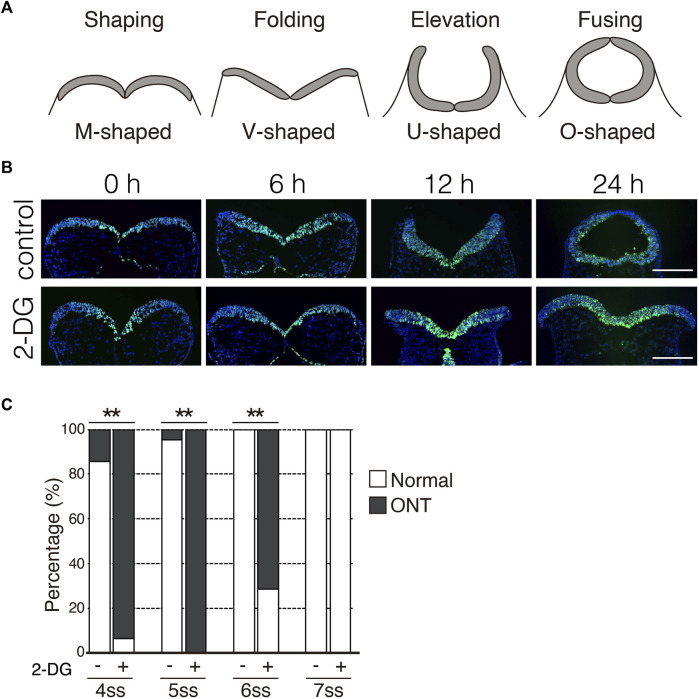
Inhibition of glycolytic activity prevents neural plate folding. **(A)** Schematic transverse sections of four stages of neural tube closure. **(B)** Neuroepithelial cells were detected by immunofluorescence staining Sox2 (green) at indicated times after incubation without (control) or with 2-DG. Scale bars, 100 μm. Images are representative of 8 (6 h in control), 9 (12 h in control), 8 (24 h in control), and 10 (6, 12, and 24 h in 2-DG) independent experiments. **(C)** Ratios (%) of embryos with normal morphology (white bar) and open neural tube (ONT, Gy bar) after incubation without (−) or with 2-DG (+) at indicated somite stage (ss). Data are shown of 14 (control, 4 somite stage), 16 (2-DG, 4 somite stage), 22 (control, 5 somite stage), 26 (2-DG, 5 somite stage), 10 (control, 6 somite stage), 14 (2-DG, 6 somite stage), 13 (control. Seven somite stage) and, 13 (2-DG 7 somite stage) embryos after incubation. Statistical differences were assessed using Chi-Square tests, **p < 0.001.

During NTC in mouse embryos, NP undergoes a dynamic morphological transformation comprising the following four spatially and temporally overlapping stages: (1) NP shaping (transverse view of NP is M-shaped), (2) NP folding (V-shaped), (3) NP elevation (U-shaped), and (4) neural fold fusion (O-shaped) (Figure 4A) (Colas and Schoenwolf, 2001). Next, we examined the stage at which the NTC is compromised by glycolytic inhibition using *exo utero* whole-embryo culture. We initially confirmed that the NTC process was precisely simulated through the appropriate steps in control cultures (Figure 4B, control) (6 h: 100%, 8/8 embryos; 12 h: 100%, 9/9 embryos; 24 h: 87.5%, 7/8 embryos). Notably, the NP retained the M shape in embryos incubated with 2-DG for 24 h (Figure 4B, 2-DG) (6 h: 100%, 10/10 embryos; 12 h: 100%, 10/10 embryos; 24 h: 100%, 10/10 embryos), suggesting that glycolytic activity is required for the onset of NP folding. The NP transformed from the M to the V shape during the first 6h in control cultures. Hence, somite segmentation is controlled by a segmentation clock with a 2-h cycles (Pourquie, 2022), when E8.0 embryos at the 3-5 somite stage develop into the 6-8 somite stage during 6 h of culture. This indicated that NP folding is sensitive to glycolytic inhibition during the 3-5 somite stage. To examine the time window of sensitivity for 2-DG exposure on NP folding, we incubated embryos with 2-DG at the 4, 5, 6, and 7 somite stages for 12, 10, 8, and 6 h, respectively. After the exposure to inhibitors, removed the inhibitors by changing the culture medium. The embryos were cultured for a further 24 h. As a result of glycolytic inhibition, 93.8% (15/16 embryos), 100% (26/26 embryos), and 71.4% (10/14 embryos) of the 4, 5, and 6 somite stage embryos, respectively, had defective NTC. Importantly, 2-DG did not affect the NTC of 7 somite stage embryos (0/13 embryos) (Figure 4C). Taken together, these results suggest that anaerobic glycolysis functions within a short time frame during neural tube development.

### Glycolytic inhibition affects cell proliferation, but not cell survival and differentiation

**FIGURE 5:**
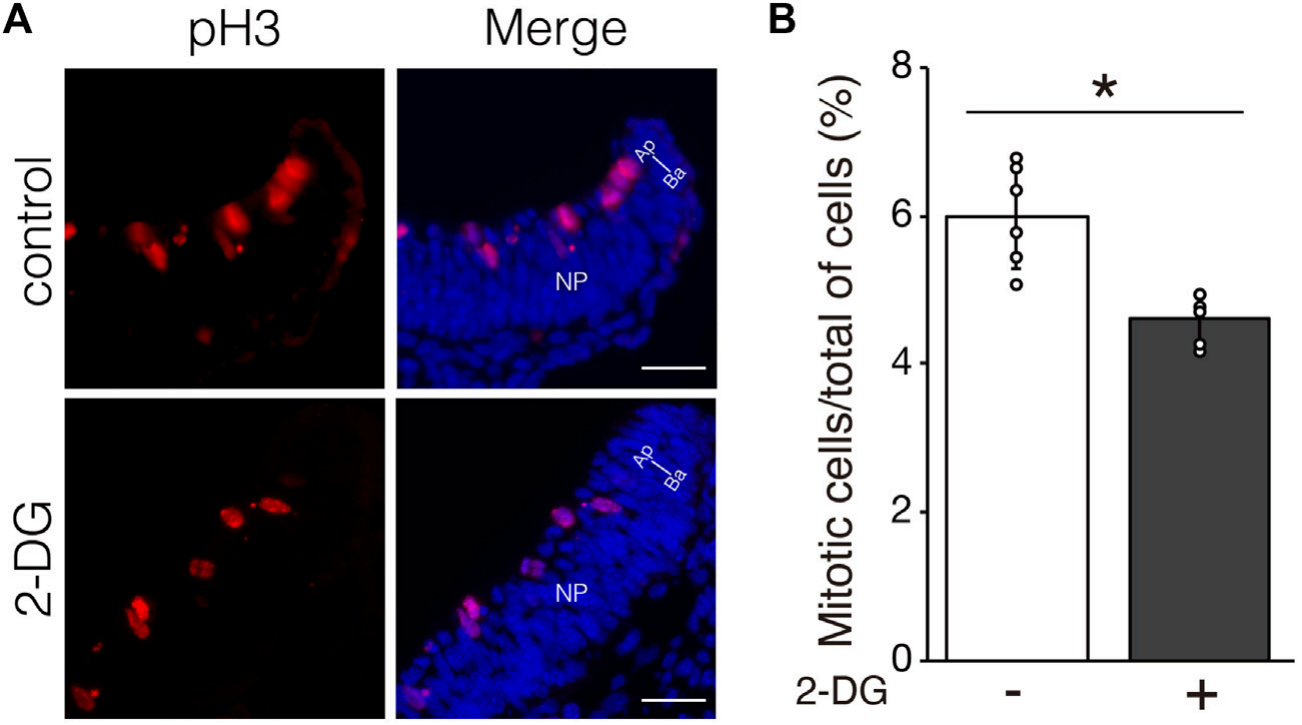
Effect of glycolytic inhibition on mitosis. **(A)** Mitotic cells detected by immunofluorescence staining phospho-histone H3 (Red) in transverse sections of the neural plate (unilateral) at E8.25 (six to eight somite stage). Directional planes are shown (Ap; apical, Ba; basal). **(B)** Percentage of phospho-histone H3^+^ mitotic cells among all neuroepithelial cells after incubation without (−) or with 2-DG (+). Data are shown as means ± S.E.M of six histological sections from three embryos. Statistical differences were assessed using Student’s *t*-tests, *p < 0.05.

We investigated whether defective NTC in 2-DG-treated embryos was associated with cell proliferation, viability, and differentiation. Proliferating and apoptotic cells were detected by immunofluorescent staining using anti-phospho-histone H3 and anti-cleaved caspase3 antibodies, respectively. The number of phospho-histone H3+ mitotic cells moderately decreased in neuroepithelial cells incubated with 2-DG (Figure 5). The numbers of apoptotic cells did not significantly differ between neuroepithelial cells incubated with or without 2-DG-treated (Supplementary Figure S1A). Cell differentiation, including dorso-ventral patterning of the NP and cranial mesenchyme formation, plays crucial roles in NTC (Yamaguchi and Miura, 2013). The expression of *Wnt1* mRNA, a marker of the dorsal edge of the NP, and *Twist1* mRNA, a marker of cranial mesenchymal cells, was not altered by 2-DG (Supplementary Figure S1B). These findings suggested that glycolysis regulates NTC through the proliferation of neuroepithelial cells.

### Glycolytic inhibition prevents apical constriction of neuroepithelial cells

**FIGURE 6:**
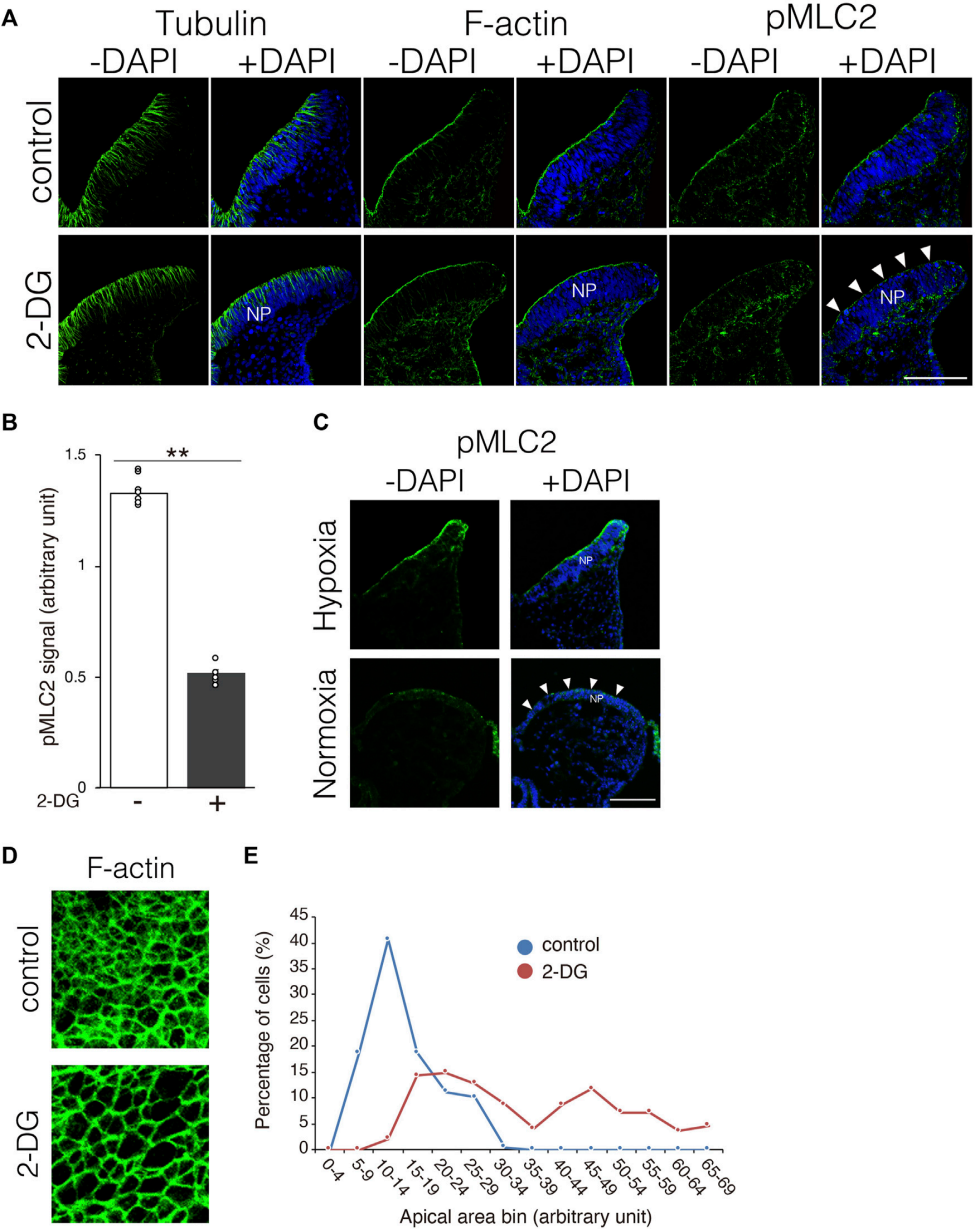
Substantially reduced pMLC2, and consequent failure of apical constriction of neuroepithelial cells caused by 2-DG. **(A)** Localization of tubulin, F-actin, and pMLC2 in transverse sections of the neural plate at E8.25 (six to eight somite stage). White arrowheads indicate reduced pMLC2 in embryos incubated with 2-DG. Scale bars, 50 μm. Images are representative of three independent experiments. White arrowheads reduced pMLC2. NP, neural plate. **(B)** Fluorescence intensity of pMLC2 in neural plate after incubation without (−) or with 2-DG (+). Data are shown as means ± S.E.M of eight histological sections from four embryos. Statistical differences were assessed using Student’s *t*-tests, **p < 0.001. **(C)** Localization of pMLC2 in transverse sections of the neural plate at E8.25 (6-8 somite stage) cultured under normoxia and hypoxia. Scale bars, 50 μm. Images are representative of eight independent experiments from four embryos. White arrowheads indicate reduced pMLC2. NP, neural plate. **(D)** F-actin rings at the apical side of NP visualized by staining with Phalloidin-488 at E8.5 (9–11 somite stage). **(E)** Graph shows numbers of neuroepithelial cells with different apical areas. Cells were counted in five histological sections from five embryos. The total cell number was 197 in control, and 195 in 2-DG, respectively. Blue, control; Red, 2-DG.

Neuroepithelial cells undergo dynamic morphological changes during the early phase of NP folding. The thickness of the NP is initially increased by the elongation of neuroepithelial cells along the apico-basal polarity, accompanied by microtubule extension. Thereafter, the apical F-actin ring constricts dependently on the activation of myosin *via* the phosphorylation of myosin light chain 2 (Suzuki et al., 2012). We investigated whether morphological changes in neuroepithelial cells are impaired by inhibiting glycolysis. Microtubule extension was comparable between NPs incubated with and without 2-DG (Figure 6A, tubulin). The F-actin ring also formed normally in NPs incubated with 2-DG (Figure 6A, F-actin). In contrast, the amount of phosphorylated myosin light chain 2 (pMLC2) was significantly reduced at the apical surface of neuroepithelial cells in 2-DG-treated embryos, suggesting impaired apical F-actin constriction (Figure 6A, pMLC2, and Figure 6B). The amount of pMLC2 was also reduced at the apical surface of neuroepithelial cells in embryos cultured under normoxia (Figure 6C). This defect is probably due to the downregulation of glycolytic gene expression in NP by normoxia (Figure 1C). We evaluated the size of the F-actin ring on the apical side of dissected phalloidin-labeled NPs using confocal microscopy. The size of the apical F-actin ring was substantially reduced in NPs incubated with 2-DG compared to NPs not incubated with 2-DG (Figure 6D). The apical area of individual cells was quantified, and its distribution was quantified. The results showed that the proportion of cells with large apical areas was significantly increased in embryos incubated with, than without 2-DG (Figure 6E). These findings indicated that glycolysis regulates myosin light chain 2 phosphorylation and subsequent apical constriction.

### Glycolytic enzymes localize at apical surfaces of neuroepithelial cells

**FIGURE 7:**
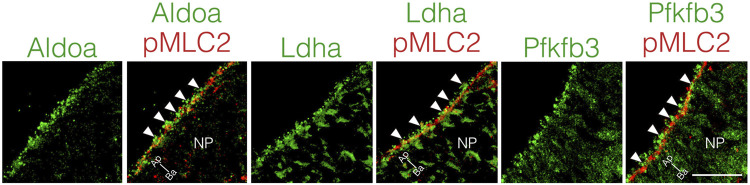
Apical localization of glycolytic enzymes in neuroepithelial cells.Localization of Aldoa, Ldha, and Pfkfb3 in transverse sections of the neural plate at E8.25 (six to eight somite stage). Apical surface visualized by pMLC2 staining. Scale bars, 20 μm. Images are representative of five independent experiments. Directional planes were shown (Ap; apical, Ba; basal). White arrowheads, punctate structures. NP, neural plate.

Local ATP synthesis contributes to the dynamic rearrangement of F-actin at the leading edge of migrating cells (De Bock et al., 2013; Cruys et al., 2016; Cunniff et al., 2016). Furthermore, some glycolytic enzymes, such as Aldoa, and Pfkfb3 protein, directly bind to F-actin, and this interaction is required for the local activation of glycolysis (Roberts and Somero, 1987; 1989; De Bock et al., 2013; Hu et al., 2016). Therefore, we examined the cellular localization of Aldoa, Ldha, and Pfkfb3 protein in neuroepithelial cells at E8.25 (six to eight somite stage). These glycolytic enzymes were localized at the apical surface of the neuroepithelial cells, where pMLC2 was also localized. Both Ldha and Pfkfb3 proteins were detected in the cytosol as well (Figure 7). Notably, Aldoa, Ldha, and Pfkfb3 proteins formed punctate structures and resided in close proximity to pMLC2; however, most of these were not colocalized (Figure 7). These results implied that ATP is locally produced to regulate apical constriction.

## Discussion

The inhibition of glycolysis by 2-DG causes NTC defects in mouse embryos (Hunter and Tugman, 1995). Similarly, the ablation of *Hif1α*, a key transcription factor for the induction of glycolytic genes under hypoxia, impairs morphogenesis in mouse embryos, including the NTC (Iyer et al., 1998; Ryan et al., 1998; Kotch et al., 1999; Compernolle et al., 2003). These findings suggest that anaerobic glycolysis is essential for NTC; however, its function in the NTC process is unknown. We investigated the role of glycolysis in detailed E8.0-E8.5 (3–11 somite stage) embryos and uncovered a critical early developmental window for NTC. We found that glycolytic activity is required for neural plate folding at a very early stage of NTC, at least until the 6 somite stage. In contrast, 7 somite stage embryos were not vulnerable to glycolysis inhibition. It has been known that embryos do not exhibit glucose metabolic plasticity before E8.5, thus anaerobic glycolysis is activated even under high oxygen concentrations (Miyazawa et al., 2017). Furthermore, glucose metabolism is rewired at E8.5; that is, glycolysis becomes coupled to the TCA cycle, and the ETC, is activated to respond to the increase in extraembryonic oxygen concentration, which is required for NP elevation (Miyazawa et al., 2018). Taking these findings into consideration, we propose that NTC can be divided according to dependence on glucose metabolism activity as NP shaping and folding that depend on anaerobic glycolysis activity (-E8.25), and subsequent NTC which depends on TCA cycle activity (E8.5-). Enhanced glycolysis plays an essential role in epithelial-mesenchymal transition during neural crest development in chick embryos (Bhattacharya et al., 2020). In mouse embryos, neural crest cells start to delaminate from NP at E8.5 (Weston et al., 2004). Thus, high levels of glycolytic activity in neuroepithelial cells may be involved in both NTC and neural crest development in mouse embryos.

Previous studies have shown that ATP is generated through anaerobic glycolysis at implantation (Zhou et al., 2012; Takashima et al., 2014; Sperber et al., 2015). The production of ATP in neuroepithelial cells relies on anaerobic glycolysis before NTC, and then the primary source of ATP switches from anaerobic glycolysis to the TCA cycle and the ETC during NTC (Miyazawa et al., 2017; Miyazawa et al., 2018; Fame et al., 2019). Taken together, these findings suggest that ATP produced by anaerobic glycolysis is involved in the early stages of NTC. What is the use of ATP during NTC? It is generally accepted that ATP provides energy to drive many cellular processes, including cell proliferation. In this study, we discovered that 2-DG reduced neuroepithelial cell proliferation (Figure 5), but had no effect on cell survival or differentiation (Supplementary Figure S1). These results indicated that anaerobic glycolysis produces ATP for neuroepithelial cell proliferation, therefore defective NTC would be partially due to impaired cell proliferation. Consistent with our findings, *Pfkfb3* promotes cell cycle progression (Jia et al., 2018). In contrast, *miR-302* ablated embryos exhibit NTC defects owing to the increased proliferation of neuroepithelial cells through upregulated glycolytic genes, including *Pfkfb3* (Keuls et al., 2020). These paradoxical findings could account for the tight control of neuroepithelial cell proliferation in normal NTC. We further demonstrated that MLC2 phosphorylation and subsequent apical constriction were substantially suppressed by glycolytic inhibition in neuroepithelial cells (Figure 6). Phosphorylation of MLC2 by Rho-associated kinase (ROCK) increases the ATPase activity of the myosin light chain (Quintin et al., 2008), and generates a contractile force for the apical constriction of neuroepithelial cells (Lee and Nagele, 1985; Rolo et al., 2009). Apical constriction reduces the sizes of the cell apices and causes morphological changes in neuroepithelial cells from rectangular to wedge-like shapes, generating a physical force for NP folding (Suzuki et al., 2012). Moreover, glycolytic enzymes have been shown in endothelial cells to regulate F-actin remodeling in filopodia and lamellipodia *via* ATP production (De Bock et al., 2013; Cruys et al., 2016). Based on our results, we believe that anaerobic glycolysis contributes to the production of ATP, which is utilized to generate physical force for NP folding. Anaerobic glycolysis is considered a less efficient metabolic process for ATP generation than the TCA cycle and the ETC Glycolytic enzymes are compartmentalized with F-actin in lamellipodia for local ATP production in endothelial cells to cope with high and rapidly changing ATP demands to maintain motor activity (De Bock et al., 2013). We found that Aldoa, Ldha, and Pfkfb3 proteins were localized at the apical surface of the neuroepithelial cells in dot-like structures located near pMLC2 (Figure 7). Thus, glycolytic enzymes might supply ATP not only for cell proliferation, but also for apical constriction, and the apical localization of enzymes might be important for the efficient supply of ATP for MLC2 phosphorylation. Whether ATP is locally generated at the apical surface of neuroepithelial cells remains unknown. Further studies are necessary to confirm local ATP generation at the apical surface by ATP imaging using an ATP biosensor.

Our principal findings were related to glycolytic inhibition by 2-DG and oxamate. Therefore, the study needs to be extended to include genetic analysis. In fact, we created neuroepithelial cell-specific *Ldha* knockout mice using *Sox1-cre* driver. These mice had normal NTC, probably due to genetic redundancy (data not shown). We also attempted to knock down *Ldha*, *Aldo*a, and *Pfkfb3* in neuroepithelial cells using a combination of siRNA electroporation and *exo utero* whole-embryo culture. However, a combination of siRNA electroporation and *exo utero* whole-embryo culture frequently induces abnormal NT development, even when an empty vector is electroporated as the control. Thus, knock down efficiency is difficult to evaluate. Further genetic studies are needed to determine the mechanism through which glycolysis regulates NTC.

## Materials and methods

### Mice

All animal experiments were performed following the Guidelines for the Care and Use of Laboratory Animals of Kanazawa Medical University. A minimum sample size of five individuals was used in each assay unless otherwise stated. ICR mice were obtained from Sankyo Lab Service. For embryonic staging, the morning on which the vaginal plug was observed was designated as E0.5.

### *Exo utero* whole-embryo culture and pharmacological inhibition of glucose metabolism

*Exo utero* whole-embryo culture was performed as previously described (Takahashi et al., 2008; Sakai et al., 2016; Ogoh et al., 2017). Figure 2A shows a schema of pharmacological inhibition. Briefly, E8.0 (three to five somite stage) embryos were dissected and cultured under a 5% O_2_; 5% CO_2_; 90% N2, 37°C atm in DR50 (50% rat serum; 50% DMEM/F-12, 2% glucose) with or without 0.1 mM 2-deoxy-D-glucose (2-DG), 28 mM oxamate, 0.1 and 0.5 mM oligomycin, or 0.1 and 0.5 mM 3-nitropropionic acid (3-NP). After 12 h of culture (corresponding to E8.5), the culture medium was changed to remove inhibitors and the cells were cultured for 24 h (corresponding to E9.5).

### RT-qPCR

We analyzed gene expression using RT-qPCR as described (Sakai et al., 2022). Briefly, total RNA extracted from E8.5 (9–11 somite stage) embryos was reverse-transcribed into cDNA, which was then amplified by qPCR using SYBR Green on a LightCycler Nano System (Roche). Gene expression was normalized to that of *Gapdh*. All samples were analyzed at least in triplicate. Relative fold change was calculated using the 2^−ΔΔCT^ method. The expression of *Aldoa, Ldha, Pfkfb3, Nqo1,* and *Gapdh* was detected using the following primers: *Aldoa* FW: TGG​GAA​GAA​GGA​GAA​CCT​GA and *Aldoa* RV: GAC​AAG​CGA​GGC​TGT​TGG; *Ldha* FW: GGCACTGACGCAGACAAG and *Ldha* RV: TGA​TCA​CCT​CGT​AGG​CAC​TG; *Pfkfb3* FW: AAC​AGC​TTT​GAG​GAG​CGT​GT and *Pfkfb3* RV: CGG​GAG​CTC​TTC​ATG​TTT​TG; *Nqo1* FW: AGC​GTT​CGG​TAT​TAC​GAT​CC and *Nqo1* RV: AGT​ACA​ATC​AGG​GCT​CTT​CTC​G; *Gapdh* FW: CAT​GTT​CCA​GTA​TGA​CTC​CAC​TC and *Gapdh* RV: GGC​CTC​ACC​CCA​TTT​GAT​GT.

### *In situ* hybridization

Some mouse *Aldoa, Ldha, and Pfkfb3* sequences were amplified by PCR using the following primers: FW-*Aldoa*: TCT​GAC​ATC​GCT​CAC​CGC​ATT and RV- *Aldoa*: AAG​AGA​GAT​TCA​CTG​GCT​GCG, FW-*Ldha*: TGA​AGA​ACC​TTA​GGC​GGG​TG and RV- *Ldha*: TGT​GTC​TCA​GAG​ACA​GTG​GG, FW-*Pfkfb3*: TCA​CCA​GGC​TGT​TCT​ACG​CT and RV- *Pfkfb3*: GTT​GTC​TTT​GCC​ACC​CCA​AC. The PCR products were cloned into the pGEM-T Easy vector (Promega) to synthesize the cRNA probes. Plasmids for the synthesis of cRNA probes against *Wnt1* and *Twist1* were gifts from Dr. Paul Trainor (Stowers Institute for Medical Research, USA).

Whole-mount *in situ* hybridization proceeded as described (Sakai et al., 2012).

### Immunofluorescence

Mouse embryos were fixed with 4% paraformaldehyde (PFA) in phosphate-buffered saline (PBS) for 3 h at 4°C and cryopreserved in 30% sucrose in PBS. Brains were embedded in optimal cutting temperature compound (OCT) and stored at −80°C until further use. The cryostat sections (10 μm thick) were adhered to glass slides and washed with PBS. Antigens were retrieved by incubation with 10 mM citric acid (pH 6.0) for 30 min at 80°C. After a brief wash with PBS, the sections were incubated with 0.5% Triton X-100 in PBS for 15 min at room temperature. Non-specific antigen binding was blocked by incubation with a blocking buffer (3% BSA in TBST) for 30 min at room temperature. The sections were then incubated at 4°C overnight with primary antibodies against phospho-histone H3 (ser28) (06-570, Upstate; 1/500), cleaved caspase 3 (9661, Cell Signaling; 1/200), Sox2 (AF 2018, R&D systems; 1/200), acetylated α-tubulin (6-11B-1)(T6793, Sigma; 1/2000), phospho-MLC2 (3671, Cell Signaling; 1/100), Aldoa (sc-12059, Santa Cruz; 1/200), Ldha (sc-27230, Santa Cruz; 1/200), and Pfkfb3 (60,241-1, Protein Tech; 1/500).

The sections were washed three times with TBST for 10 min and then incubated with the appropriate secondary antibodies conjugated with Alexa 488 or 546 (A11001, A21208, Invitrogen; 1/300) for 1 h at room temperature. Nuclei were stained with DAPI. Sections were assessed using a BX51 fluorescence microscope equipped with a DP30BW CCD camera (Olympus) and 10× and 20× objective lenses. Images were acquired using the DP controller software (Olympus).

### Apical surface area calculations

We outlined apical membranes by staining the F-actin ring with Alexa Fluor-488 phalloidin (A12379, Thermo Fisher; 1/1000). Sections were assessed using a LSM PASCAL confocal fluorescence microscope (Carl Zeiss) and a 40× objective lens. Confocal optical slices were collected, and maximum-intensity projections of 0.3 mm stacks were generated using Zeiss LSM5 software. The apical surface area was calculated using ImageJ software.

### Statistics

Data were statistically analyzed using two-tailed Student's *t*-tests and Chi-square test. Values with *p* < 0.05 indicated statistically significant differences.

## Data Availability

The datasets generated during current study are available in the Figshare, at 10.6084/m9.figshare.23577999.

## Supplementary material

SUPPLEMENTARY FIGURE S1 Effect of glycolytic inhibition on apoptosis and differentiation. **(B)** Percentage of cleaved caspase3^+^ apoptotic cells among all neuroepithelial cells after incubation without (-) or with 2-DG (+). Data are shown as means ± S.E.M of six histological sections from three embryos. Statistical differences were assessed using Student’s *t*-tests. **(C)** Expression of *Wnt1,* and *Twist1* mRNA was detected by cryosection *in situ* hybridization of E8.25 embryos. Scale bars, 100 μm. Image is representative of four independent experiments.
