# Supplementary figures and images for: Glycolytic activity is required for the onset of neural plate folding during neural tube closure in mouse embryos
Source: Front Cell Dev Biol. 2023 Jul 3;11:1212375. doi: 10.3389/fcell.2023.1212375 (PMC10350492; doi:10.3389/fcell.2023.1212375)

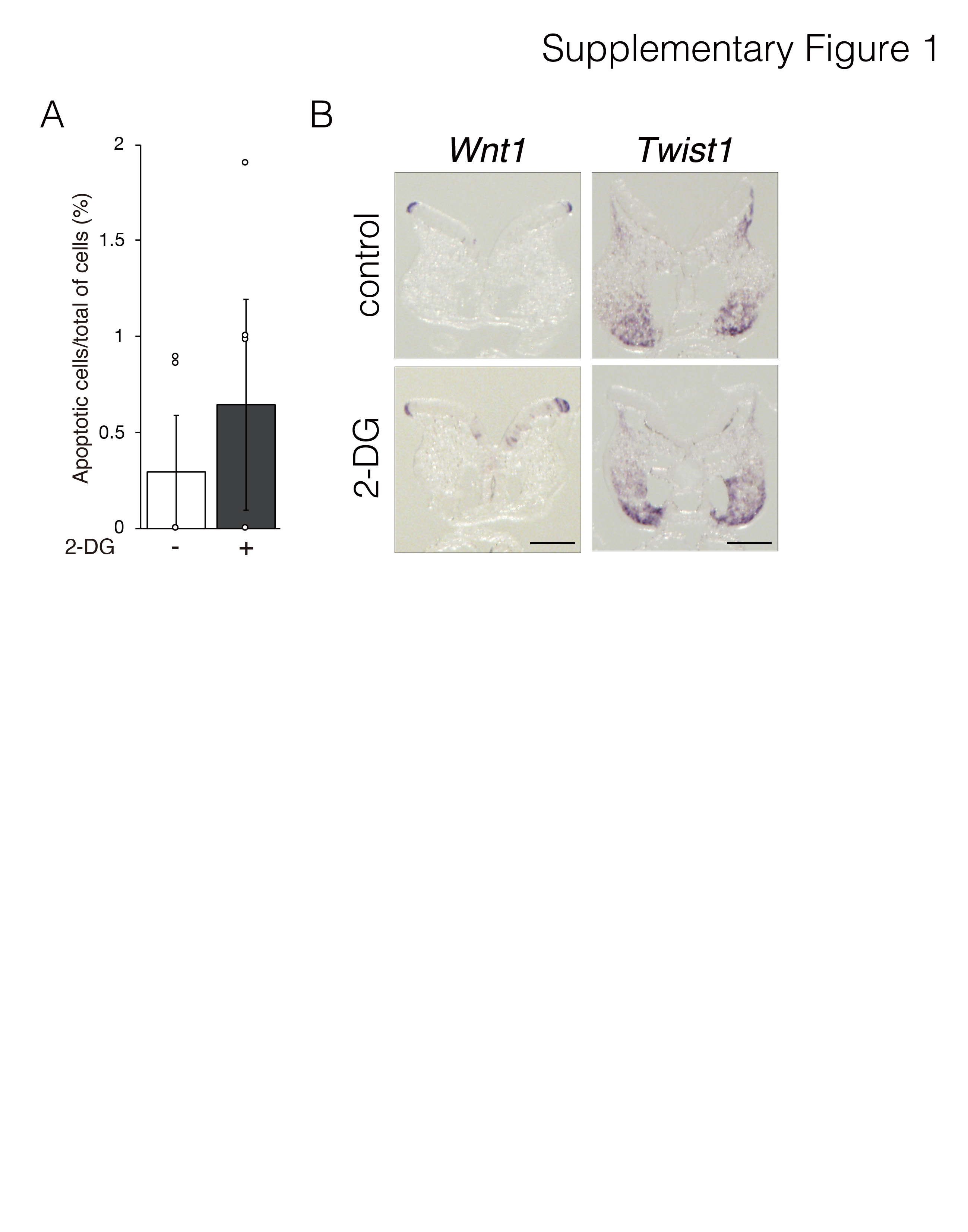

Supplement: Supplementary file 1 [file Image1.JPEG]
